# Hodgkin Lymphoma Involving the Brain: Report of a Rare Case

**DOI:** 10.7759/cureus.52173

**Published:** 2024-01-12

**Authors:** Muhammad Tahir, Osama Elkadi, Jacek Polski

**Affiliations:** 1 Pathology and Laboratory Medicine, University of South Alabama Health Hospital, Mobile, USA; 2 Anatomical and Clinical Pathology, University of South Alabama Health Hospital, Mobile, USA

**Keywords:** hodgkin lymphoma, hodgkin lymphoma of the brain, anti-cd30, relapse lymphoma, hodgkin lymphoma involving the brain

## Abstract

Hodgkin lymphoma (HL) is a systemic neoplastic process that mostly affects the lymph nodes of the cervical, supraclavicular, and mediastinal regions. The involvement of the central nervous system by HL is rare, and the diagnosis is typically determined based on distinct morphological and immunohistochemical analyses performed on tissue biopsy specimens. Intracranial HL is so scarce that any of the intracranial space-occupying pathology in a patient with established HL must be meticulously investigated to rule out a secondary disease process. Here, we report a unique case of intracranial HL in a 31-year-old African American male who was previously treated with anti-CD30 antibodies.

## Introduction

Hodgkin lymphoma (HL) is a systemic disease that usually affects lymph nodes, with a primary extra-nodal presentation of HL being quite rare. The most involved lymph nodes are the cervical, mediastinal, and supraclavicular. The involvement of the central nervous system (CNS) by primary or metastatic HL is extremely rare, previously reported as occurring in 0.2% to 0.5% of the patients with systemic HL [1]. Central nervous system involvement by HL is usually associated with severe systemic relapse of the disease. The most commonly and universally accepted treatment options for HL involving the brain are local surgical excision followed by targeted radiation and systemic chemotherapy [2].

This article was previously submitted as an abstract and presented as a poster at the annual American Society of Clinical Pathologists (ASCP) 2023 conference on October 18th, 2023, in Long Beach, California.

## Case presentation

A 31-year-old African American male with a past medical history of attention deficit hyperactivity disorder was admitted with a primary concern of severe hypercalcemia in the setting of months of dry cough and B symptoms, including night sweats and weight loss.

Initial diagnostic workup and treatment for hypercalcemia, including computed tomography (CT) of the chest, bronchoscopy with endobronchial ultrasound, pulmonary function test, parathyroid hormone, calcitriol, granulomatous disease workup, including acid-fast bacilli, rapid plasma protein, human immunodeficiency virus, bronchoalveolar lavage, calcitonin, and significant intravenous fluid with diuresis, were completed. After extensive clinical and laboratory workup with no significant clinical improvement, the patient was referred for a hematology and oncology consultation for the possibility of Hodgkin lymphoma versus other etiologies of mediastinal mass and hypercalcemia in malignancy. A CT of the chest and abdomen was performed and was significant for mediastinal/subcarinal lymph adenopathy as well as significant splenomegaly.

**Figure 1:**
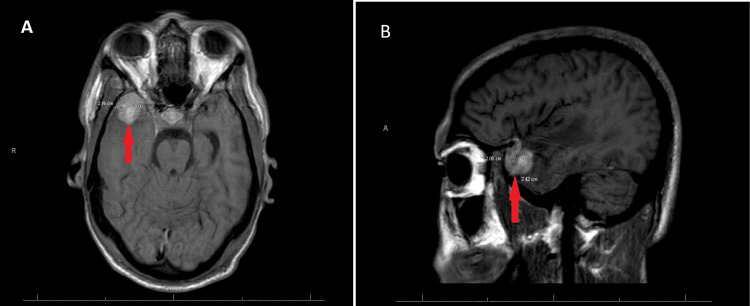
MRI of the brain in coronal and sagittal views (Panels A and B, respectively), with red arrows indicating the lesion at the right anterior temporal pole MRI: magnetic resonance imaging

Following mediastinal station 7 lymph node biopsy and histopathological exanimation, the patient was diagnosed with classical Hodgkin lymphoma and was started on an Adriamycin, vinblastine sulfate, dacarbazine (AVD), and brentuximab regimen. One month after the initial diagnosis, the patient presented to the emergency department with neurological symptoms, and magnetic resonance imaging (MRI) of the brain showed a spherical lesion near the gray-white matter differentiation of the right anterior temporal pole (Figure 1). The lesion was homogenous and had a peripheral diffusion restriction. The lesion measured 1 x 1 x 1.1 cm. There was also a significant amount of surrounding vasogenic edema. The MRI finding favored a right temporal lobe neoplasm with a differential diagnosis that includes astrocytoma versus metastasis. A surgical resection was planned, and two weeks later, the mass was resected.

**Figure 2:**
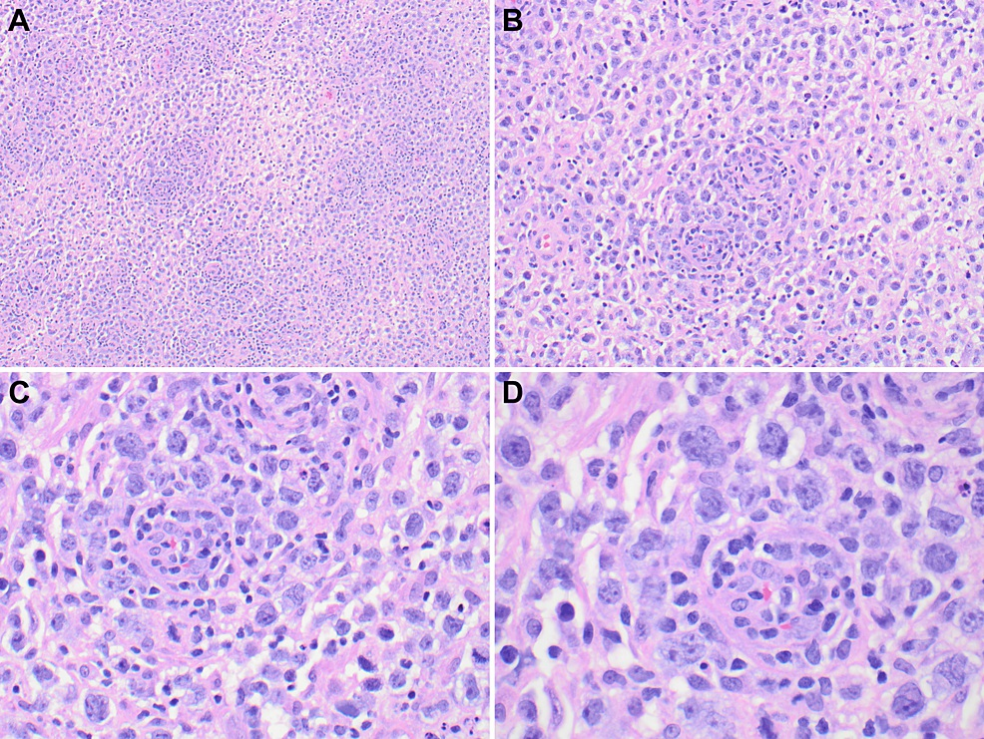
Low and medium power views show sheets of atypical, pleomorphic neoplastic cells in a background of mixed inflammatory cells (Panels A and B, 4x and 10x, respectively). High-power views showing the typical Reed-Sternberg cells (Panels C and D, 20x and 40x, respectively)

Histologically, the resection specimen consists of sheets of atypical, pleomorphic cells. Most of these large, atypical-looking cells have a bilobated to multilobated nucleus, a prominent eosinophilic nucleolus, and ample amphophilic cytoplasm consistent with classic Reed-Sternberg cells. There are numerous large and atypical mononuclear cells similar to those seen in nodal, so-called syncytial Hodgkin cells (Figure 2).

**Figure 3:**
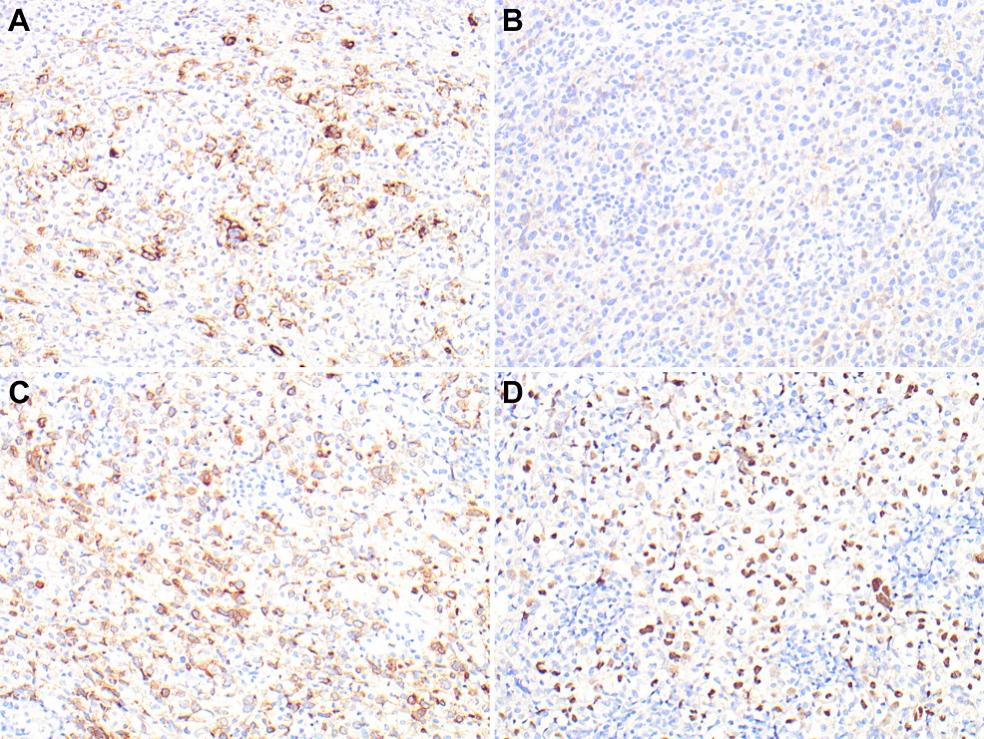
CD15 immunostaining is diffusely positive in neoplastic Reed-Sternberg cells (Figure A, 20x). CD30 is weak or partial due to anti-CD30 treatment (Panel B. 20x). CD79a and PAX5 are diffusely positive in neoplastic cells (Panels C and D, 20x). CD: cluster of differentiation, PAX5: paired box 5, B-cell lineage specific activator protein

Multiple immunohistochemical stains were used for the final characterization of the disease. The classic large Reed-Sternberg cells are positive for CD15, CD30 (partial and weak), CD79a, and PAX5 (Figure 3). The weak expression of CD30 was attributed to the history of treatment with anti-CD30 antibodies. The malignant cells were mostly negative for CD3, CD10, CD45, Bcl-2, ALK1, MUM-1, and SV40. A diagnosis of classic HL was rendered based on histology and immunohistochemical profiling.

## Discussion

HL is a malignancy originating in the lymphatic system and characterized by the presence of Reed-Sternberg cells. Although HL typically involves lymph nodes, it can rarely metastasize to distant sites, including the brain. As summarized by a recent review, only 45 cases of CNS HL have been reported from 2000 to 2018 [1]. The involvement of the brain by HL often presents with nonspecific neurological symptoms such as headaches, seizures, cognitive impairments, personality changes, and focal neurological deficits. These symptoms can be challenging to differentiate from other brain metastases or primary brain tumors. Therefore, a high index of suspicion is crucial, especially in patients with a history of HL or refractory disease [2,3].

The diagnosis of HL involving the brain requires a comprehensive evaluation. Initially, a thorough clinical examination and detailed medical history review are essential. Neuroimaging techniques, including MRI with gadolinium enhancement, provide valuable information regarding the location, size, and number of brain lesions. Additionally, a cerebrospinal fluid (CSF) examination may be performed to detect malignant cells and assess tumor-related biomarkers [4]. When CSF shows limited cellularity, the newly available diagnostic techniques such as molecular testing by using circulating tumor DNA (ctDNA or cfDNA) can be helpful diagnostic tools [5].

The management of HL involving the brain requires a multidisciplinary approach involving oncologists, neurologists, neurosurgeons, and radiation oncologists. The treatment strategy depends on several factors, including the extent of brain involvement, the patient's overall health status, and the response to previous treatments. Therapeutic options may include a combination of chemotherapy, radiation therapy, targeted therapies, and surgery for symptomatic or accessible lesions [6].

Chemotherapy, often tailored to the specific patient and disease characteristics, is a fundamental component of the treatment regimen. High-dose methotrexate-based regimens, along with other agents, such as cytarabine, etoposide, or ifosfamide, have shown efficacy in controlling brain metastases. Radiation therapy, either whole-brain irradiation or stereotactic radiosurgery, may be employed to target specific brain lesions or residual disease after systemic therapy. Targeted therapies, such as monoclonal antibodies or immune checkpoint inhibitors, may also be considered in select cases [7].

Refractory or relapsed classical Hodgkin lymphoma (HL) treatment remains challenging nevertheless targeted immunotherapy has recently emerged as a promising treatment option for these patients. CD30 is overexpressed in a variety of lymphomas, including Hodgkin lymphoma, several peripheral T-cell lymphomas, and some cutaneous T-cell lymphomas. Brentuximab vedotin, an antibody-drug combination that targets CD30-positive cells, has been investigated for the treatment of various lymphoma types [5,6]. Although first-generation monoclonal anti-CD30 antibodies were not satisfactory, contemporary efforts to modify anti-CD30 antibodies in order to enhance the binding of effector cells and maximize activity, as well as the development of novel antibody-drug conjugates (ADCs), appear more promising. ADCs provide the capability to deliver potent medications with very little toxicity. Brentuximab vedotin (SGN-35) is a highly active ADC that incorporates an anti-CD30 monoclonal antibody with the anti-tubulin agent monomethyl auristatin E. Preliminary phase 1 studies of brentuximab vedotin in recurrent HL demonstrated a 52% overall response rate with very low toxicity [6-9]. In our case, the patient was treated with anti-CD30 antibodies for primary HL. Partial membranous CD30 staining, with loss of intense staining attributed to treatment effect by brentuximab.

The prognosis of HL involving the brain is poor due to its aggressive nature and the presence of disseminated disease. Overall survival depends on various factors, including the extent of systemic disease, the number and size of brain lesions, and the response to treatment. Despite advancements in therapy, the presence of brain metastases remains a significant challenge, as they often indicate advanced and refractory disease [2].

## Conclusions

CNS involvement in HL is rare, but it must be considered for the patient with a previous history of HL who presented with the newly developed intracranial lesion. For diagnosis, proper advanced radiological techniques should be used, which will be helpful in complicated cases with unusual radiological presentations. The need for tissue diagnosis is essential in all patients with a previous history of HL who present with unexplained clinical neurological symptoms and focal mass-forming CNS lesions. A multidisciplinary approach is needed for these cases because there is no consensus on the best treatment now, and surgery followed by radiotherapy and intrathecal chemotherapy should be considered on an individual basis and according to the complexity of each case. At present, the prognosis for this rare complication is poor and needs to be improved and optimized.
